# The clinical relevance of ABO blood type in 100 patients with acute subdural hematoma

**DOI:** 10.1371/journal.pone.0204331

**Published:** 2018-10-04

**Authors:** Daniel Dubinski, Sae-Yeon Won, Bedjan Behmanesh, Nina Brawanski, Christof Geisen, Volker Seifert, Christian Senft, Juergen Konczalla

**Affiliations:** 1 Department of Neurosurgery, University Hospital, Goethe University, Frankfurt, Germany; 2 Institute for Transfusion Medicine and Immunohematology, Goethe University, Frankfurt, Germany; Roswell Park Cancer Institute, UNITED STATES

## Abstract

**Objective:**

The correlation of depleted blood through midline shift in acute subdural hematoma remains the most reliable clinical predictor to date. On the other hand, patient’s ABO blood type has a profound impact on coagulation and hemostasis. We conducted this study to evaluate the role of patient’s blood type in terms of incidence, clinical course and outcome after acute subdural hematoma bleeding.

**Methods:**

100 patients with acute subdural hematoma treated between 2010 and 2015 at the author’s institution were included. Baseline characteristics and clinical findings including Glasgow coma scale, Glasgow outcome scale, hematoma volume, rebleeding, midline shift, postoperative seizures and the presence of anticoagulation were analyzed for their association with ABO blood type.

**Results:**

Patient’s with blood type O were found to have a lower midline shift (p<0.01) and significantly less seizures (OR: 0.43; p<0.05) compared to non-O patients. Furthermore, patients with blood type A had the a significantly higher midline shift (p<0.05) and a significantly increased risk for postoperative seizures (OR: 4.01; p<0.001). There was no difference in ABO blood type distribution between acute subdural hematoma patients and the average population.

**Conclusion:**

The ABO blood type has significant influence on acute subdural hematoma sequelae. Patient’s with blood type O benefit in their clinical course after acute subdural hematoma whereas blood type A patients are at highest risk for increased midline shift and postoperative seizures. Further studies elucidating the biological mechanisms of blood type depended hemostaseology and its role in acute subdural hematoma are required for the development of an appropriate intervention.

## Introduction

Isolated acute subdural hematoma (aSDH) is a form of intracranial bleeding defined by the presence of a blood collection between the dura and arachnoid layer, that requires immediate intensive treatment and the mortality rates are as high as 50–80%. [1] [2] Despite the presence of several outcome prediction models that are based on clinical parameters such as the initial Glasgow coma scale (GCS), present anticoagulation therapy, pupillary status and the time between onset and operation, the prediction of patient’s outcome remains challenging. [3] [4] [5] [6] Midline-shift on the other hand is an established parameter that is associated with a higher frequency of unfavourable outcome and death. [7] [8] Accordingly, additional risk factors are required for the prediction of short and long-term prognosis in these patients. In recent years several studies described the profound influence of the ABO blood type system on patients haemostasis and thrombosis. [9,10] The ABO blood type is determined by the glycosyltransferase on chromosome 9 that attaches monosaccharaides to the cell membrane on erythrocytes, platelets and vascular endothelial cells. The blood type O is a result of an inactive glycosyltransferase and is furthermore strongly associated with a reduction of the circulating procoagulatory vWF (von Willebrand factor) and F VIII (Factor VIII) plasma level of up to 30% compared to non-O blood type. [11] [12] As vWF binds and transports F VIII, this interaction is vital for platelet/matrix binding in terms of haemostatic response to vascular injury. A decreased plasma level of vWF and F VIII in patients with blood type O is therefore of direct clinical significance. [13][14] Coherent clinical studies confirmed the blood type O as a bleeding risk factor in several disciplines. [15][9] In line, patients with non-O blood type were shown to have an increased risk for suffering from VTE (venous thromboembolism), coronary heart disease and arterial thrombosis. [16,17] The influence of patients ABO blood type on clinical course and outcome after aSDH has not been investigated yet. We conducted this study to explore the role of patients ABO blood type as a new risk/prognostic factor.

## Material and methods

### Patients and data collection

The present study was approved by the clinical ethics committee of the University of Frankfurt. All patients over 18 years old with the diagnosis of isolated aSDH admitted to our University Hospital from 2010 to 2015 were identified retrospectively using the electronic database. All data were fully anonymized before access; hence no informed consent was required. Exclusion criterions included the lack of radiological data or ABO blood type, traumatic brain injury (TBI), pre-existing hematological disorders or hospital discharge in less than 24 hours after admission. Investigated medical record parameters included: ABO blood type, age at admission, gender, hematoma volume by ABC/2 formula, [18] midline shift, GCS at admission, GCS 24 hours after surgical treatment, rebleeding rate, GOS at 3 months, occurrence of postoperative seizures and anticoagulation treatment.

The radiological examinations were performed using the initial CT scan. For volume measurements the estimating formula ABC/2 was used. In patients with bilateral hematomas, both volumes were added together. For measurement of the midline shift and the width of the hematoma, the maximum diameter was used. In cases of clinically suspected epileptic seizures, we assessed the surface EEG recording that was reviewed by experienced neurologists who were blind to the patient’s outcome. Patient’s functional outcome was evaluated using the Glasgow Outcome Scale (GOS) at 3 months follow-up.

### Study design

The present analysis is a retrospective, single centre observational study of isolated aSDH patients. The aim of the study was (1) to observe the incidence of the ABO blood type compared to the general German population, (2) examine the possible influence of ABO blood type on preoperative parameters and (3) to correlate the ABO blood type with patient’s outcome.

### Statistics

Data analysis was performed with BiAS (Version 11.06.2017). For parametric parameters, the ANOVA test was used. For nonparametric parameters, the Wilcoxon-Mann-Whitney test was used. To assess the impact of the variables, odds ratio (OR) with 95% confidence intervals (CI) was calculated. Results with p ≤0.05 were considered statistically relevant.

## Results

**Fig 1:**
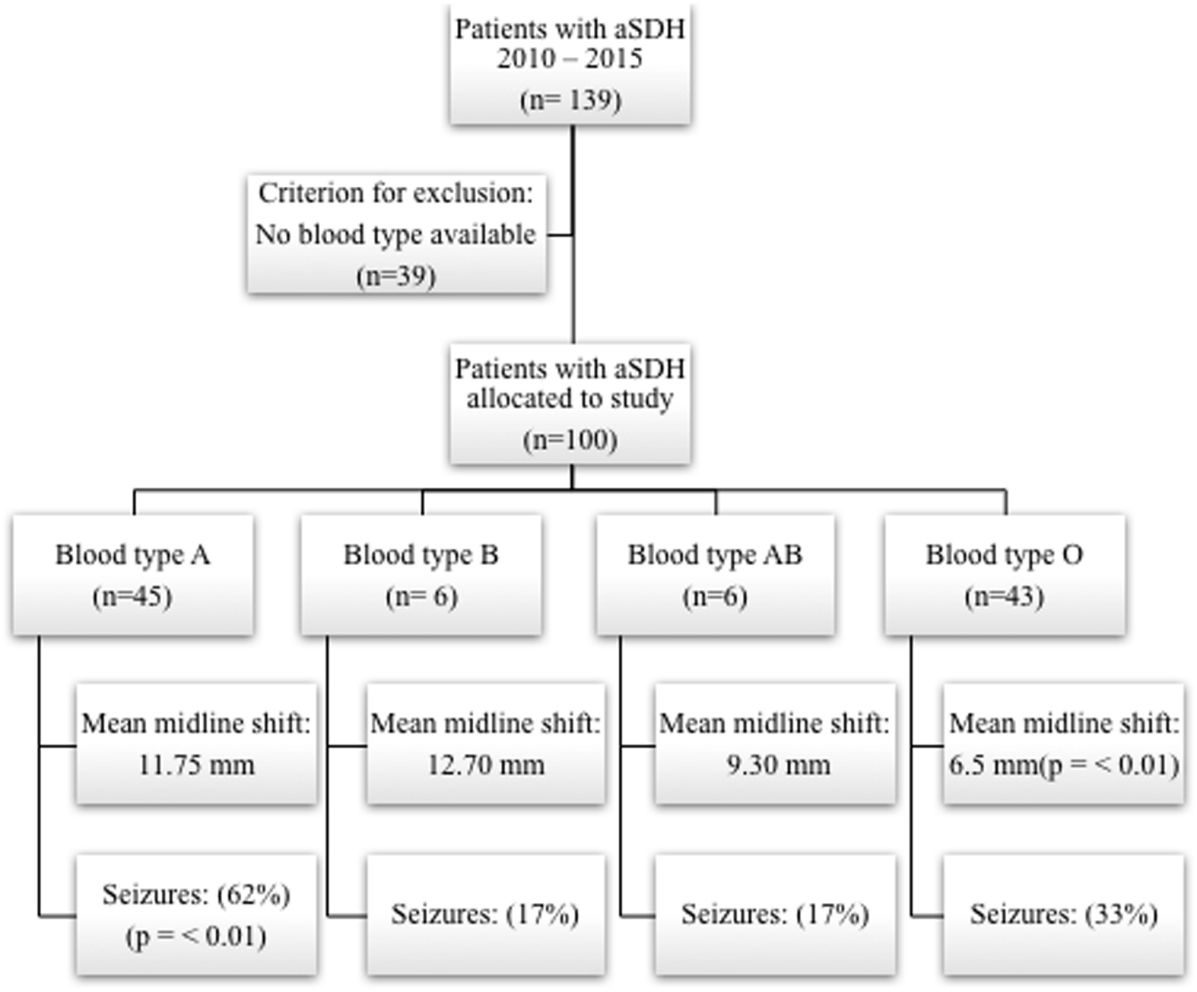
Study allocation with the illustration of major findings: Blood type A patients are at higher risk for postoperative seizures whereas patients with blood type O benefit in their clinical course after aSDH.

A total of 139 patients were eligible for this study. ABO blood type was not available in 39 cases. Thus, 100 patients (mean age 79 years, 33 female) were included in this study (study allocation displayed in Fig 1). Mean aSDH volume measured in the initial CT scan at admission was 105.16 ± 37.96 mm^3^ and a midline shift of 11.25 mm ± 5.14 mm. There was an even distribution between left and right hematoma side (47 vs. 49) and in 4 cases bilateral aSDH was diagnosed. Mean GCS at admission was 7.25 ± 3.87, postoperative 3.75 ± 5.02 and 7.75 ± 4.81 at discharge. 20% of the patients suffered from rebleeding and postoperative seizures were recorded in 44%. Mean GOS 3 months after discharge was 3 ± 1.61. 72% of the patients presented with anticoagulation at admission. For detailed patient’s baseline characteristics separated by ABO blood group see Table 1, S1 Table.

**Table 1:** 

Blood Group, *n (%)*	All, 100 *(100%)*	A, 45 *(45%)*	*P*-value *(A vs*. *non-A)*	OR *[95% CI]*	B, 6 *(6%)*	*P*-value *(B vs*. *non-B)*	OR *[95% CI]*	AB, 6 *(6%)*	*P*-value *(AB vs*. *non-AB)*2	OR *[95% CI]*	O, 43 *(43%)*	*P*-value *(O vs*. *non-O)*	OR *[95% CI]*
Sex (female), *n (%)*	33 (33)	15 (33)	n.s.	n.s.	3 (50)	n.s.	n.s.	3 (50)	n.s.	n.s.	12 (28)	n.s.	n.s.
Age (mean), *n (SD)*	79 (13.91)	80 (13.25)	n.s.	n.s.	78.50 (9.70)	n.s.	n.s.	77.50 (17.33)	n.s.	n.s.	79 (14.88)	n.s.	n.s.
Mean GCS (preoperative), *(SD)*	7.25 (3.87)	7 (3.81)	n.s.	n.s.	7.5 (3.93)	n.s.	n.s.	11.5 (3.74)	**0.051**	[7.00–11.50]	6 (4.13)	n.s.	n.s.
Mean GCS (postoperative), *(SD)*	3.75 (5.02)	3.0 (4.30)	n.s.	n.s.	4.5 (4.27)	n.s.	n.s.	11.5 (5.42)	n.s.	n.s.	3.0 (5.11)	n.s.	n.s.
Mean GCS (discharge), *(SD)*	7.75 (4.81)	8 (5.25)	n.s.	n.s.	12 (4.14)	n.s.	n.s.	7.5 (4.57)	n.s.	n.s.	6 (4.92)	n.s.	n.s.
Mean GOS (3 month), *(SD)*	3 (1.61)	3 (1.73)	n.s.	n.s.	3 (1.50)	n.s.	n.s.	1.3 (0.81)	**0.018**	[3.00–0.50]	3 (1.91)	n.s.	n.s.
Mean volume (cm3), *(SD)*	105.16 (37.96)	111.78 (54.10)	n.s.	n.s.	140.87 (39.96)	n.s.	n.s.	76.46 (34.12)	n.s.	n.s.	98.55 (35.97)	n.s.	n.s.
Mean midline shift (mm), *(SD)*	11.25 (5.14)	11.75 (7.02)	**0.029**	[7.20–11.85]	12.70 (2.37)	n.s.	n.s.	9.30 (4.77)	n.s.	n.s.	6.50 (5.52)	**0.007**	[6.50–11.75]
Rebleeding, *n (%)*	20 (20)	8 (18)	n.s.	n.s.	2 (33)	n.s.	n.s.	3 (50)	n.s.	n.s.	7 (16)	n.s.	n.s.
Seizure, *n (%)*	44 (44)	28 (62)	**0,0008**	4.01 [1.73–9.27]	1 (17)	n.s.	n.s.	1 (17)	n.s.	n.s.	14 (32)	**0.035**	0.43 [0.19–0.98]
Anticoagulation (at admission), *n (%)*	72 (72)	34 (76)	n.s.	n.s.	5 (83)	n.s.	n.s.	6 (100)	n.s.	n.s.	27 (63)	n.s.	n.s.

Detailed patients baseline characteristics separated by ABO blood group. Blood type O patients benefit in their clinical course, whereas blood type A patients are at higher risk for aSDH sequel.

### ABO blood type and the prevalence of aSDH

**Fig 2:**
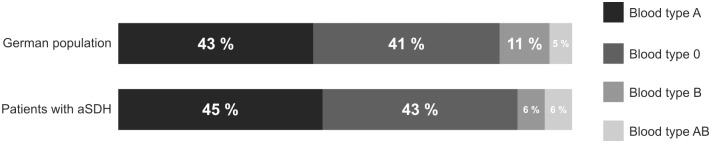
Comparison of the ABO blood type distribution among the analysed cohort and German population.

45 patients carry the blood type A (45%), followed by 43 patients with blood type O (43%), 6 patients with the AB blood type (6%) and 6 patients with blood type B (6%). There was no significant difference in blood type distribution between our analyzed cohort and ABO blood type distribution among the German population. The ABO distribution among German population and aSDH patients is displayed in Fig 2.

### Advantage of blood type O in the clinical course of patients with aSDH

Postoperative seizures are strongly associated with poor outcome. In our cohort 14 (32%) patients with blood type O had epileptic seizures. This was the case for 28 (62%) of the patients with blood type A, one patient (17%) with blood type B, and one patient (17%) with the AB blood type. Therefore, patients with blood type O had a significantly decreased risk in the development of postoperative epileptic seizures (OR 0.43; 95% CI [0.09–0.98]; p = 0.035) (Table 2).

**Table 2:** 

Characteristics	All, 100 *(100%)*	seizure, *n = 44*	no seizure, *n = 56*	*P*-value	OR *[95% CI]*
Sex (female), *n (%)*	33 (33)	13 (30)	20 (36)	n.s.	n.s.
Age (mean), *n (SD)*	79 (13.91)	79 (13.41)	79 (14.41)	n.s.	n.s.
Blood type A, *n (%)*	45 (45)	30 (68)	15 (27)	**0,0001**	5.85 [2.46–13.94]
Blood type O, *n (%)*	43 (43)	14 (32)	29 (52)	**0.035**	0.43 [0.19–0.98]
Blood type B, *n (%)*	6 (6)	1 (2)	5 (9)	n.s.	n.s.
Blood type AB, *n (%)*	6 (6)	1 (2)	5 (9)	n.s.	n.s.
Mean GCS (preoperative), *(SD)*	7.25 (3.87)	6 (3.48)	9 (4.25)	**0,0003**	3.78 [1.42–4.57]
Mean GCS (postoperative), *(SD)*	3.75 (5.02)	3 (4.41)	6 (5.16)	**0.002**	3.07 [1.06–4.93]
Mean GCS (discharge), *(SD)*	7.75 (4.81)	3 (4.97)	11 (4.63)	**0,0001**	8.30 [6.08–9.91]
Mean GOS (3 month), *(SD)*	3 (1.61)	3 (1.44)	3 (1.69)	n.s.	n.s.
Mean volume (cm3), *(SD)*	105.16 (37.96)	99.31 (45.43)	93.15 (50.98)	n.s.	n.s.
Mean midline shift (mm), *(SD)*	9.1 (5.14)	11.3 (6.73)	8.7 (5.79)	**0,04**	2.07 [0.11–5.08]
Rebleeding, *n (%)*	20 (20)	7 (16)	13 (23)	n.s.	n.s.
Anticoagulation (at admission), *n (%)*	72 (72)	37 (84)	35 (63)	n.s.	n.s.

Allocation of the investigated cohort according to the onset of postoperative seizures. Significant increase was observed for patients with blood type A, whereas blood type O patients were at lower risk for the development of postoperative seizures.

**Fig 3:**
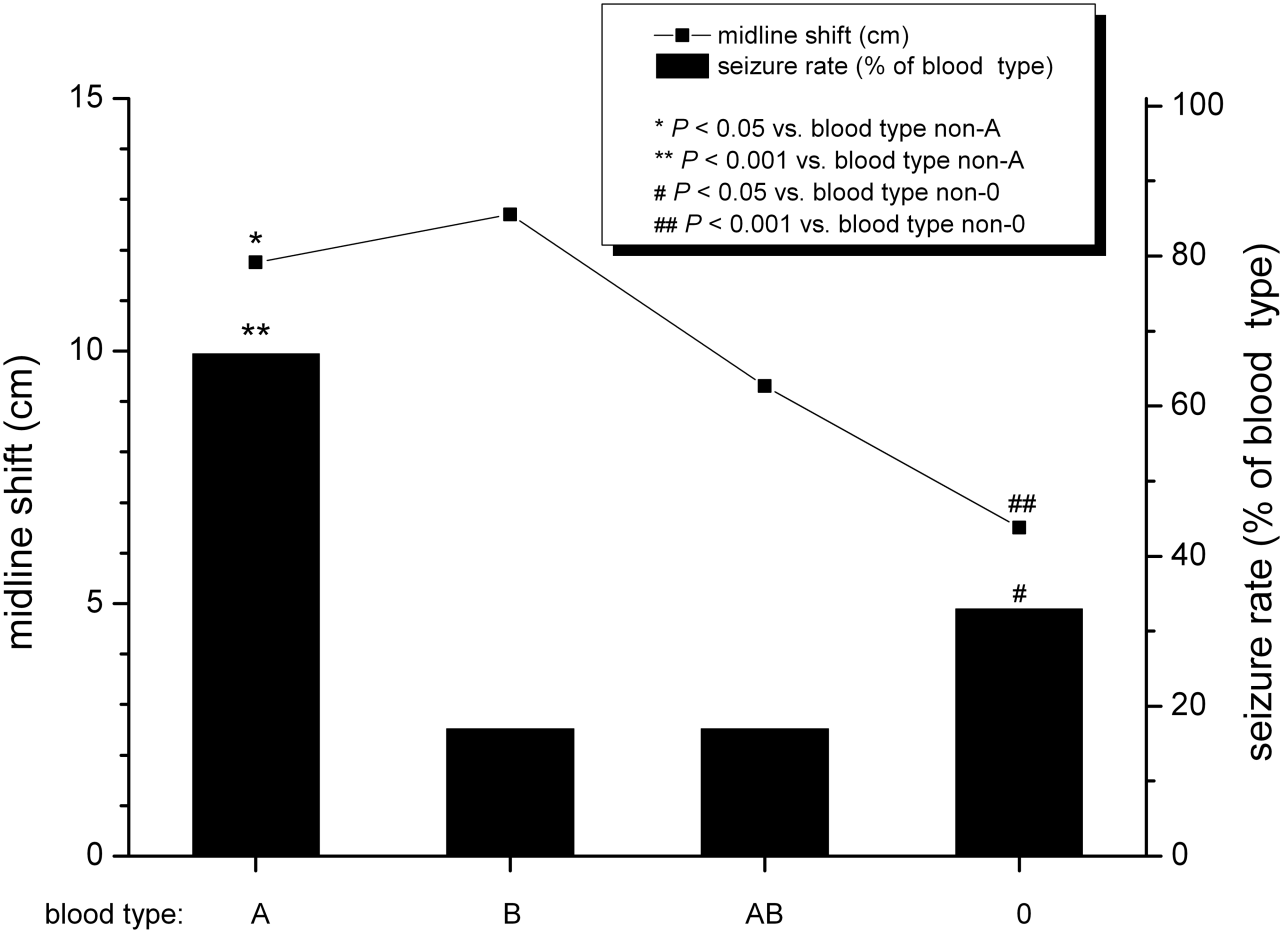
Patients allocated according to the ABO blood type. The left ordinate shows the mean midline shift of patients with aSDH and. The right ordinate shows the percentage of patients with postoperative seizures.

The preoperative midline shift was measured on the initial CT-scan as mentioned in the material section. Patients with blood type O had a mean midline shift of 6.5 ± 5.52 mm, blood type A patients had a mean midline shift of 11.7 ± 7 mm, blood type AB patients had a mean midline shift of 9.30 ± 4.77 mm whereas blood type B patients had 12.7 ± 2.37 mm. A significant lower midline shift was observed in blood type O patients (p = 0.007; median range 11.75–6.5 mm). (Table 1, Fig 3).

### Blood type A is associated with an increased risk for aSDH sequelae

Among the analyzed parameters, the occurrence of postoperative seizures (verified via EEG as described in the methods section) was investigated. Statistical analysis showed a significant increase s in blood type A patients for the development of postoperative epileptic seizures (OR 4.01; 95% CI [1.73–9.27]; p = 0.0008).

Further, blood type A patients had a mean midline shift of 11.75 ± 7.02 mm in the preoperative CT scan. We found this parameter significantly increased compared to the other ABO blood types. (p = 0.029; median range 7.20–11.85 mm). (Table 1 Fig 3).

### Patient’s present anticoagulation therapy does not influence aSDH volume, midline shift and outcome

To exclude the bias role of anticoagulation therapy in the comparison between the ABO blood types, we allocated the cohort according to present anticoagulation. Anticoagulation was present in significantly older patients (OR 6.18; 95% CI [11.55–22.45]; p = 0.0001) and was associated with poor postoperative outcome represented via GCS (OR 5.48; 95% CI [-8.17–3.82]; p = 0.0001) and at discharge (OR 5.48; 95% CI [3.82–8.17]; p = 0.0001) respectively. However, the presence of anticoagulation did not influence the mean subdural blood volume (OR 0.60; 95% CI [14.80–27.82]; p = 0.545), the mean midline shift (OR 1.88; 95% CI [0.12–5.33]; p = 0.561), the rebleeding rate (OR 1.21; 95% CI [0.39–3.71]; p = 0.789) nor the outcome analyzed through the GOS 3, month post-operative (OR 1; 95% CI [2.59–4.39]; p = 1). (See Table 3, Fig 3).

**Table 3:** 

Characteristics	All, 100 *(100%)*	Anticoagulation, *n = 72*	No anticoagulation, *n = 28*	*P*-value	OR *[95% CI]*
Sex (female), *n (%)*	33 (33)	24 (33)	9 (32)	n.s.	n.s.
Age (mean), *n (SD)*	79 (13.91)	81 (10.74)	64 (17.12)	**0.0001**	6.18 [11.55–22.45]
Blood type A, *n (%)*	45 (45)	34 (47)	11 (39)	n.s.	n.s.
Blood type B, *n (%)*	6 (6)	5 (7)	1 (4)	n.s.	n.s.
Blood type AB, *n (%)*	6 (6)	6 (8)	0 (0)	n.a	n/a
Blood type O, *n (%)*	43 (43)	27 (37.5)	16 (57)	n.s.	n.s.
Mean GCS (preoperative), *(SD)*	7.25 (3.87)	6.5 (3.96)	8 (4.12)	n.s.	n.s.
Mean GCS (postoperative), *(SD)*	3.75 (5.02)	3 (4.80)	9 (5.19)	**0,0001**	5.48 [3.82–8.17]
Mean GCS (discharge), *(SD)*	7.75 (4.81)	6 (4.81)	12 (5.18)	**0,0001**	5.48 [3.82–8.17]
Mean GOS (3 month), *(SD)*	3 (1.61)	3 (1.54)	3 (10.92)	n.s.	n.s.
Mean volume (cm3), *(SD)*	105.16 (37.96)	93.70 (47.98)	100.03 (48.83)	n.s.	n.s.
Mean midline shift (mm), *(SD)*	9.1 (5.14)	9.8 (6.50)	7.2 (5.66)	n.s.	n.s.
Rebleeding, *n (%)*	20 (20)	15 (21)	5 (18)	n.s.	n.s.

Splitting cohort allocation according to present anticoagulation therapy to exclude its bias role. Apart from age and in hospital GCS, no significant difference was observed.

### The influence of patients ABO blood type on clinical course and functional outcome

Patients with blood type O that were found to have a decreased midline shift and lower postoperative epileptic seizure incidence had interestingly no significant difference in the initial admission status represented through the GCS score, compared to non-O patients (p = 0.997; mean GCS 6). This effect remained to be not significant in the postoperative GCS evaluation (p = 0.727; mean GCS 3) and at discharge (p = 0.605; mean GCS 6; see Table 1). In terms of rebleeding rate no significant difference between O and non-O patients was found either (OR 0.65; 95% CI [0.23–1.82]; p = 0.291). Regarding the morbidity outcome expressed through the GOS at 3 months, no significant difference could be found between O and non-O patients either. (p = 0.504 mean GOS 3). Interestingly patients with AB blood type had a borderline significant better initial admission status expressed in a higher GCS score (p = 0.051 mean GCS 11.5) compared to non-AB patients. This effect was not detectable in the postoperative nor in the GCS status at discharge p = 0.269 and p = 0.666 respectively. Surprisingly GOS analysis at 3 months showed a significantly lower outcome in AB patients compared non-AB. (p = 0.018 mean range 3.00–0.50).

## Discussion

Our analysis focused on the association between ABO blood type and the clinical course of aSDH patient’s. We identified several parameters that were significantly dependent on patient’s ABO blood type and describe our findings as additional new risk factors in aSDH burden.

aSDH is a serious condition that can cause mass effect, midline shift, and brain herniation. Prognostic factors are therefore highly needed in the clinical setting for predicting patient’s outcome and their clinical course. Several prognostic factors were described to be associated with poor outcome in aSDH including midline shift, initial poor GCS, seizures and antithrombotic medication. [4,8,19] On the other hand recent studies defined patients ABO blood type as a genetic bleeding risk factor. Clinical investigations and epidemiological data showed that patient’s with blood type O were at higher risk for clinically relevant hemorrhages, presumably due to the lowest amount of procoagulatory proteins in this blood group. [9,12,20] Vice versa non-O blood type patients were identified to be at an increased risk for developing VTE, coronary heart disease and arterial thrombosis. [17,21,22]

Interestingly, the Midline shift was significantly decreased in blood type O patients compared with non-O patients, presumably due to faster clot liquidation. Furthermore, the potential bias of anticoagulation therapy could be excluded in our analysis, which strengthen the clinical influence of ABO blood type in aSDH.

Counter-intuitively ABO blood type did not influence the postoperative rebleeding rate among our cohort. This finding could be in part explained by the rapid hemostaseologic interventions during patient’s intensive care treatment in terms of coagulation factor substitution and postoperative anticoagulation necessity.

Postoperative epileptic seizures are an important risk factor in aSDH contributing to patients’ morbidity and mortality. Whereas patients with blood type O had a significantly decreased risk for seizure development supposable through different clot properties and thereby lower cortex irritation, patients with blood type A had a significantly increased risk for postoperative seizures. So, especially patients with blood type A should receive a postoperative EEG.

In our cohort, ABO blood type did not significantly influence patient outcome evaluated through GCS score at admission, neither during intensive care treatment, nor at discharge. However, patients with the AB blood type (bearing the highest amount of procoagulatory proteins) showed the worst long-term outcome evaluated through the GOS scale at 3 months. Hemostaseological properties related to hematoma liquidation, patients’ recovery and rehabilitation progress could be responsible for this finding. Therefore, further analysis should prospectively identify embolic complications during follow-up period.

Today’s understanding of patients ABO blood type role in hemosatseology and thrombosis is gained mainly from extra-cranial cases. However, aSDH is a serious condition with high morbidity and mortality rates, especially among the elderly. [23,24] As the population ages, the prevalence of aSDH and patients with anticoagulation therapy is anticipated to identify the need for individualized hemostaseologic therapy, reliable risk and prognostic factors. [23,24]

To the best of our knowledge, data regarding the ABO blood type system and the clinical course after aSDH hemorrhage has not been investigated to date. Therefore, our data adds new insights in aSDH hemostaseology. However, our study has certain limitations. The present analysis was performed retrospective that is a subject for several bias itself. Further, several patients had to be excluded due to the lack of blood type data, making a selection bias possible. However, patient’s characteristics and a general blood group distribution indicate a good representation in our cohort. Furthermore, three patients were operated as an “outpatient procedure” (missing operative capacity in other hospitals with neurosurgical department or missing own ICU capacity in our hospital). In these patient’s also a lot of data is missing—especially history of anticoagulation, so these patients were excluded. Last to mention, data regarding long-term outcome further than 3 months after incidence was not included, excluding the possibility of the ABO blood type as a long-term outcome factor after aSDH.

## Conclusion

To date several established risk factors for poor outcome after aSDH are available. However, as the morbidity and mortality rates after aSDH are high, precise outcome factors are urgently needed for treatment recommendation, especially among the elderly. Here, we present the patients ABO blood type as a genetic risk factor with direct clinical implication. Patients with blood type A are at a significantly high risk for increased midline shift and postoperative seizures. So, we recommend a postoperative EEG to identify seizure and to treat these patients adequately. The biological mechanisms of aSDH sequelae related to the ABO blood type need to be investigated further.

## Supporting information

S1 Table Patients characteristics. Clinical parameters including ABO blood type.
(XLSX)

## References

[1] Servadei F. Prognostic factors in severely head injured adult patients with acute subdural haematoma’s. Acta Neurochir (Wien). Springer-Verlag; 1997;139: 279–285. 10.1007/BF01808822 9202766

[2] Phuenpathom N, Choomuang M, Ratanalert S. Outcome and outcome prediction in acute subdural hematoma. Surg Neurol. 1993;40: 22–5. Available: http://www.ncbi.nlm.nih.gov/pubmed/8322172 8322172 10.1016/0090-3019(93)90164-v

[3] Kotwica Z, Brzeziński J. Acute subdural haematoma in adults: an analysis of outcome in comatose patients. Acta Neurochir (Wien). 1993;121: 95–99. 10.1007/BF01809257 8512021

[4] Klun B, Fettich M. Factors influencing the outcome in acute subdural haematoma. A review of 330 cases. Acta Neurochir (Wien). 1984;71: 171–8. Available: http://www.ncbi.nlm.nih.gov/pubmed/6741634 6741634 10.1007/BF01401312

[5] Leitgeb J, Mauritz W, Brazinova A, Janciak I, Majdan M, Wilbacher I, et al Outcome after severe brain trauma due to acute subdural hematoma. J Neurosurg. 2012;117: 324–333. 10.3171/2012.4.JNS111448 22631691

[6] Senft C, Schuster T, Forster M-T, Seifert V, Gerlach R. Management and outcome of patients with acute traumatic subdural hematomas and pre-injury oral anticoagulation therapy. Neurol Res. 2009;31: 1012–1018. 10.1179/174313209X409034 19570326

[7] Jacobs B, Beems T, van der Vliet TM, Diaz-Arrastia RR, Borm GF, Vos PE. Computed Tomography and Outcome in Moderate and Severe Traumatic Brain Injury: Hematoma Volume and Midline Shift Revisited. J Neurotrauma. 2011;28: 203–215. 10.1089/neu.2010.1558 21294647

[8] Bartels RH, Meijer FJ, van der Hoeven H, Edwards M, Prokop M. Midline shift in relation to thickness of traumatic acute subdural hematoma predicts mortality. BMC Neurol. 2015;15: 220 10.1186/s12883-015-0479-x 26496765 PMC4620003

[9] Dentali F, Sironi AP, Ageno W, Bonfanti C, Crestani S, Frattini F, et al Relationship between ABO blood group and hemorrhage: a systematic literature review and meta-analysis. Semin Thromb Hemost. 2013;39: 72–82. 10.1055/s-0032-1329550 23299820

[10] Dentali F, Sironi AP, Ageno W, Crestani S, Franchini M. ABO blood group and vascular disease: an update. Semin Thromb Hemost. 2014;40: 49–59. 10.1055/s-0033-1363460 24381150

[11] Zhang H, Mooney CJ, Reilly MP. ABO Blood Groups and Cardiovascular Diseases. Int J Vasc Med. 2012;2012: 1–11. 10.1155/2012/641917 23133757 PMC3485501

[12] Zabaneh D, Gaunt TR, Kumari M, Drenos F, Shah S, Berry D, et al Genetic Variants Associated with von Willebrand Factor Levels in Healthy Men and Women Identified Using the HumanCVD BeadChip. Ann Hum Genet. 2011;75: 456–467. 10.1111/j.1469-1809.2011.00654.x 21534939

[13] TERRAUBE V, O’DONNELL JS, JENKINS P V. Factor VIII and von Willebrand factor interaction: biological, clinical and therapeutic importance. Haemophilia. 2010;16: 3–13. 10.1111/j.1365-2516.2009.02005.x 19473409

[14] Lau CS, McLaren M, Belch JJ. Factor VIII von Willebrand factor antigen levels correlate with symptom severity in patients with Raynaud’s phenomenon. Br J Rheumatol. 1991;30: 433–6. Available: http://www.ncbi.nlm.nih.gov/pubmed/1747698 1747698 10.1093/rheumatology/30.6.433

[15] Kahr MK, Franke D, Brun R, Wisser J, Zimmermann R, Haslinger C. Blood group O: A novel risk factor for increased postpartum blood loss? Haemophilia. 2018; 10.1111/hae.13537 29877601

[16] Dentali F, Sironi AP, Ageno W, Turato S, Bonfanti C, Frattini F, et al Non-O blood type is the commonest genetic risk factor for VTE: results from a meta-analysis of the literature. Semin Thromb Hemost. 2012;38: 535–48. 10.1055/s-0032-1315758 22740183

[17] He M, Wolpin B, Rexrode K, Manson JE, Rimm E, Hu FB, et al ABO blood group and risk of coronary heart disease in two prospective cohort studies. Arterioscler Thromb Vasc Biol. 2012;32: 2314–20. 10.1161/ATVBAHA.112.248757 22895671 PMC3488453

[18] Won S-Y, Zagorcic A, Dubinski D, Quick-Weller J, Herrmann E, Seifert V, et al Excellent accuracy of ABC/2 volume formula compared to computer-assisted volumetric analysis of subdural hematomas. PLoS One. 2018;13 10.1371/journal.pone.0199809 29944717 PMC6019668

[19] Servadei F, Nasi MT, Giuliani G, Cremonini AM, Cenni P, Zappi D, et al CT prognostic factors in acute subdural haematomas: the value of the “worst” CT scan. Br J Neurosurg. 2000;14: 110–6. Available: http://www.ncbi.nlm.nih.gov/pubmed/10889882 10889882 10.1080/02688690050004525

[20] Wu O, Bayoumi N, Vickers MA, Clark P. ABO(H) blood groups and vascular disease: a systematic review and meta-analysis. J Thromb Haemost. 2008;6: 62–9. 10.1111/j.1538-7836.2007.02818.x 17973651

[21] Streiff MB, Segal J, Grossman SA, Kickler TS, Weir EG. ABO blood group is a potent risk factor for venous thromboembolism in patients with malignant gliomas. Cancer. 2004;100: 1717–23. 10.1002/cncr.20150 15073862

[22] Streiff MB, Ye X, Kickler TS, Desideri S, Jani J, Fisher J, et al A prospective multicenter study of venous thromboembolism in patients with newly-diagnosed high-grade glioma: hazard rate and risk factors. J Neurooncol. 2015;124: 299–305. 10.1007/s11060-015-1840-z 26100546 PMC4659392

[23] Won S-Y, Dubinski D, Brawanski N, Strzelczyk A, Seifert V, Freiman TM, et al Significant increase in acute subdural hematoma in octo- and nonagenarians: Surgical treatment, functional outcome, and predictors in this patient cohort. Neurosurg Focus. 2017;43 10.3171/2017.7.FOCUS17417 29088952

[24] Won S-Y, Dubinski D, Bruder M, Cattani A, Seifert V, Konczalla J. Acute subdural hematoma in patients on oral anticoagulant therapy: management and outcome. Neurosurg Focus. 2017;43: E12 10.3171/2017.8.FOCUS17421 29088960

